# Elimination of formate production in *Clostridium thermocellum*

**DOI:** 10.1007/s10295-015-1644-3

**Published:** 2015-07-11

**Authors:** Thomas Rydzak, Lee R. Lynd, Adam M. Guss

**Affiliations:** Biosciences Division, Oak Ridge National Laboratory, Oak Ridge, TN USA; BioEnergy Science Center, Oak Ridge National Laboratory, One Bethel Valley Road Oak Ridge, Tennessee, 37831-6038 USA; Thayer School of Engineering at Dartmouth College, Hanover, NH USA

**Keywords:** Cellulosic ethanol, *Clostridium thermocellum*, Pyruvate:formate lyase, Metabolic engineering, C1 metabolism

## Abstract

The ability of *Clostridium thermocellum* to rapidly degrade cellulose and ferment resulting hydrolysis products into ethanol makes it a promising platform organism for cellulosic biofuel production via consolidated bioprocessing. Currently, however, ethanol yield is far below theoretical maximum due to branched product pathways that divert carbon and electrons towards formate, H_2_, lactate, acetate, and secreted amino acids. To redirect carbon and electron flux away from formate, genes encoding pyruvate:formate lyase (*pflB*) and PFL-activating enzyme (*pflA*) were deleted. Formate production in the resulting Δ*pfl* strain was eliminated and acetate production decreased by 50 % on both complex and defined medium. The growth rate of the Δ*pfl* strain decreased by 2.9-fold on defined medium and biphasic growth was observed on complex medium. Supplementation of defined medium with 2 mM formate restored Δ*pfl* growth rate to 80 % of the parent strain. The role of *pfl* in metabolic engineering strategies and C_1_ metabolism is discussed.

**Electronic supplementary material:**

The online version of this article (doi:10.1007/s10295-015-1644-3) contains supplementary material, which is available to authorized users.

## Introduction

Growing global energy demands, rural economic development, the volatile cost of fossil fuels, and environmental concerns have prompted research into the development of sustainable and environmentally benign energy sources. Biofuels provide a promising alternative to petroleum-derived fuels for transportation, one of the largest and fastest growing energy sectors [6]. Currently, bioethanol is a leading candidate that can be used with current technologies as a fuel supplement or replacement. A number of strains that produce ethanol at high yield and titer (e.g., *Saccharomyces cerevisiae* or *Zymomonas mobilis*) are available for industrial bioethanol production, but require monosaccharides or disaccharides typically derived from food/feed sources (e.g., corn, beets, sugarcane). These sugars can also be generated through chemical or enzymatic hydrolysis of highly abundant lignocellulosic biomass. However, consolidated bioprocessing (CBP), featuring one-step processing without added enzymes, has potential for lower costs as compared to processes that involve a dedicated step for cellulase production [23–25]. While a number of organisms are capable of cellulase-mediated cellulose hydrolysis and subsequent fermentation to ethanol, none have been yet identified or developed that can produce ethanol at high yields and titer required for commercial production.

**Fig. 1:**
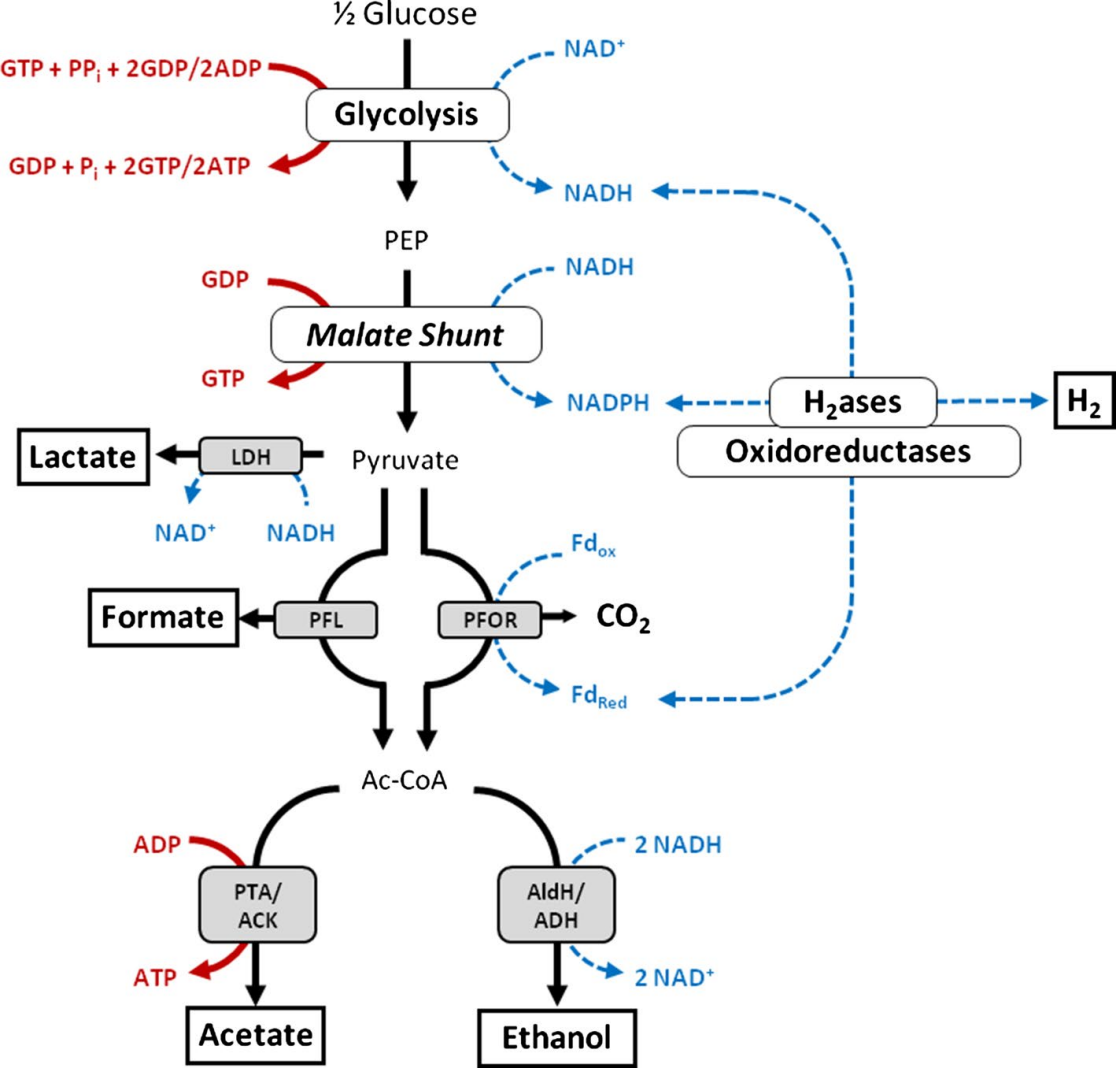
Fermentation pathways in *C. thermocellum*. *Fd* ferredoxin; *LDH* lactate dehydrogenase; *PFOR* pyruvate: Fd oxidoreductase; *PFL* pyruvate: formate lyase; *PTA/ACK* phosphotransacetylase/acetate kinase; *AldH/ADH* aldehyde dehydrogenase/alcohol dehydrogenase; *H*_*2*_ *ases* hydrogenases including ech-type Fd-dependent hydrogenase and bifurcating hydrogenases. Oxidoreductases include NADH dependent reduced ferredoxin: NADP^+^ oxidoreductase (NfnAB) and NADH:Fd oxidoreductase (RNF)

*Clostridium thermocellum* is a promising candidate for ethanol production via CBP given its inherent ability to rapidly solubilize cellulose and ferment the hydrolysis products to biofuels (i.e., ethanol and H_2_) [13, 21, 22]. However, branched metabolic pathways divert carbon and/or electrons away from ethanol towards undesired fermentation products including formate, H_2_, lactate, and acetate (Fig. 1). Many of these pathways have been elucidated through enzymology [15, 20, 28, 32, 37, 38, 44], transcriptomics [5, 29, 42, 43], proteomics [30, 31, 34], and genetics [1, 3, 7, 39]. More recent studies have demonstrated that carbon and electron flux are also diverted towards secreted amino acids [8, 14, 41] and other compounds including pyruvate, malate, fumarate, isobutanol, and butanediol, [12], further limiting ethanol yields.

While a number of studies have demonstrated that manipulation of fermentation conditions can influence product yields [5, 16, 33], engineering of a *C. thermocellum* strain capable of high-yield ethanol production will require the elimination of the pathways involved in production of competing fermentation products. Recent advances related to genetic tools applicable to *C. thermocellum*, including electrotransformation protocols [27, 40], positive and negative selection systems used to select for gene deletions [1, 39], and improvement of transformation efficiencies through elimination of Dcm methylation of plasmid DNA [9] have allowed for genetic engineering of *C. thermocellum*. These tools have been used to begin engineering *C. thermocellum* to increase ethanol yield. Previous deletion of genes involved in many competing pathways have included phosphotransacetylase (*pta*; [1, 39]), lactate dehydrogenase (*ldh*; [1, 2]), malate dehydrogenase (*mdh*; [7]), malic enzyme (*malE*; [7]), Ech-type ferredoxin-dependent hydrogenase (*ech*; [3]), and a [FeFe] hydrogenase maturation factor to inactivate [FeFe] hydrogenases (*hydG*; [3]). Further, to increase NADH availability for bifunctional aldehyde/alcohol dehydrogenase (*adhE*), heterologous expression of pyruvate kinase was used to bypass the ‘malate shunt’ to reduce transhydrogenation that generates NADPH from using electrons from NADH [7], and expression of a mutant AdhE allele was also shown to increase ethanol tolerance in *C. thermocellum* [4].

While most pathways to products other than ethanol have been deleted in *C. thermocellum*, this is not the case for formate synthesis. Production of reduced compounds such as formate limits the electrons available for ethanol production; therefore, identifying and deleting the gene(s) involved in formate synthesis are important next steps in the engineering of *C. thermocellum* for high-yield ethanol production for CBP.

In this study, we simultaneously deleted pyruvate:formate lyase (*pflB*; Clo1313_1717) and Pfl-activating enzyme (*pflA*; Clo1313_1716) in *C. thermocellum* in an attempt to increase electron flux towards ethanol and to understand how this mutation impacts growth, end-product synthesis, and amino acid secretion on rich and defined medium.

## Materials and methods

### Strains, culture conditions, and reagents

*Saccharomyces cerevisiae* InvSc1 (uracil auxotroph; Life Technologies, Grand Island, NY), used for yeast gap repair cloning, was maintained on YPD medium and grown on SD-ura medium (Sunrise Science Products, San Diego, CA) when selecting for presence of URA3^+^ plasmids. *Escherichia coli* Top10 (*dam*^+^*dcm*^+^; Invitrogen, Carlsbad, CA) and BL21 (*dam*^+^*dcm*^−^; New England Biolabs, Ipswich, MA) strains were used for plasmid construction and propagated aerobically on LB medium supplemented with 12 μg/ml chloramphenicol as required for plasmid maintenance. *Clostridium thermocellum* strains were derived from strain DSM 1313 (Deutsche Sammlung von Microorganismen und Zellkulturen, Braunshwieg, Germany) and were routinely grown anaerobically at 55 °C, unless otherwise noted, in a Coy anaerobic chamber (Coy Laboratory Products, Grass Lake, MI) on 5 g/l cellobiose in modified DSM 122 complex medium supplemented with 50 mM MOPS and 10 mM sodium citrate [39] (referred to as CTFUD). Medium was made anaerobic via autoclaving to remove O_2_ from solution, followed by immediate transfer to the anaerobic chamber to maintain anaerobicity and was supplemented with 10 μg/ml thiamphenicol, 50 μg/ml 5-fluoro-2′-deoxyuridine, or 500 μg/ml 8-azahypoxanthine (Tokyo Chemical Industry, Co., Tokyo, Japan) during *C. thermocellum* strain construction when appropriate. *C. thermocellum* strains were grown in either CTFUD or a modified version of chemically defined Medium for Thermophilic Clostridia (MTC; [36]) in which (i) urea concentrations were reduced and medium was supplemented with trace elements and additional vitamins as outlined by [2] and (ii) all stock solutions were filter sterilized rather than autoclaved. This modified version of MTC is now referred to as ‘MTC_5_’. Tubes containing either CTFUD or MTC_5_ were made anaerobic by degassing/gassing (2:1 min) ten times with 100 % N_2_. Final pressure of tubes was equilibrated to 5 psi over ambient pressure. All chemicals were reagent grade and obtained from Sigma-Aldrich (St. Loius, MO) or Fisher Scientific (Waltham, MA) unless otherwise specified.

### Plasmid and strain construction

All plasmids used in this study were constructed using yeast gap repair cloning as outlined by Shanks et al. [35]. Plasmid isolation and purification were performed using QIAprep spin miniprep kits (QIAGEN, Germantown, MD). *C. thermocellum* Δ*hpt* [1] was transformed via electroporation as previously described [9, 27]. The *pflAB* genes were deleted according to the protocol developed in Argyros et al. [1] and detailed in Olson et al. [27] using plasmid pAMG281 (Accession number KP864661; Online Resource 1). Gene deletion was confirmed using primer sets P1_F_ (5′-GAAATTATACTCCTTATGAAGGCGA-3′) and P1_R_ (5′-TCTGTTCCTTGACTGCTGCAA-3′); P2_F_ (5′-CGGAGCCCAACCTTACAGTAC-3′) and P2_R_ (5′-TATGGAAAGGGTCGGAGTGG-3′); and P3_F_ (5′-ATACTTGATTATTATGAGCGCGG-3′) and P3_R_ (5′-ATTCTCCTGGTTAAGCCTTGTAA-3′) as described below.

### Fermentation conditions

Fermentation experiments were carried out in sealed Balch tubes (27 ml; Belco Glass Inc., Vineland, NJ) containing either CTFUD or MTC_5_ as described above. *C. thermocellum* inoculum was subcultured a minimum of three times using a 2 % (v/v) inoculum on corresponding medium to prevent carry over. Fermentations were performed on 4.5 g/l cellobiose at 55 °C until all substrate was consumed. Final fermentation products were measured following complete cellobiose utilization (<0.25 mM remaining). Fermentations were performed a minimum of two times with three independent biological replicates each time. For growth curves, cells were grown in 650 μl total volume and growth was monitored spectrophotometrically at OD_600_ in an Eon Microplate Spectrophotometer (BioTek Instruments Inc., Winooski, VT) situated in a Coy anaerobic chamber.

### Analytical methods

Substrate (cellobiose), the cellobiose hydrolysis product glucose, and fermentation products (pyruvate, lactate, acetate, formate, ethanol) were analyzed using a Breeze High Performance Liquid Chromatography system (Waters, Milford, MA) using an Aminex-HPX-87H column (Bio-Rad, Hercules, CA) with a 5 mM sulfuric acid mobile phase. H_2_ was measured using an Agilent Technologies 6850 Series II Gas Chromatograph (Agilent Technologies, Santa Clara, CA) using a thermoconductivity detector set at 190 °C with an N_2_ reference flow and a Carbonex 1010 PLOT (30.0 m × 530 μm I.D.; model Supelco 25467) column. Secreted amino acids were measured using an Aracus Amino Acid Analyzer (membraPure, Berlin, Germany) using a T111 Li-cation exchange column with eluents supplied by the manufacturer. Ninhydrin-derived amino acids were measured photometrically at 570 nm with the exception of proline, which was measured at 440 nm. Final pH was measured using an Accument AB15 Basic pH meter (Fisher Scientific; Pittsburg, PA).

### Calculations

CO_2_ produced was calculated based on the expected ratio of C_1_:C_2_ compounds and the fact that valine biosynthesis also liberates CO_2_, whereby CO_2_ = [(ethanol + acetate) − (formate)] + valine. Ratios of oxidized to reduced fermentation products (O/R) were calculated using reduction values of each fermentation product, calculated as the number of oxygen atoms less one-half the number of hydrogens in each compound [26]. Carbon bound electron equivalents were calculated as described by Harris et al. [10] and were used to determine electron recovery.

## Results

### Deletion of *C. thermocellumpfl* eliminates formate and decreases acetate production

The genes encoding pyruvate-formate lyase (*pflB*) and Pfl-activating enzyme (*pflA*) were deleted in *C. thermocellum* Δ*hpt*, confirmed using three primer sets as described in Online Resource 2, and is hereafter referred to as *C. thermocellum* Δ*pfl*.

**Fig. 2:**
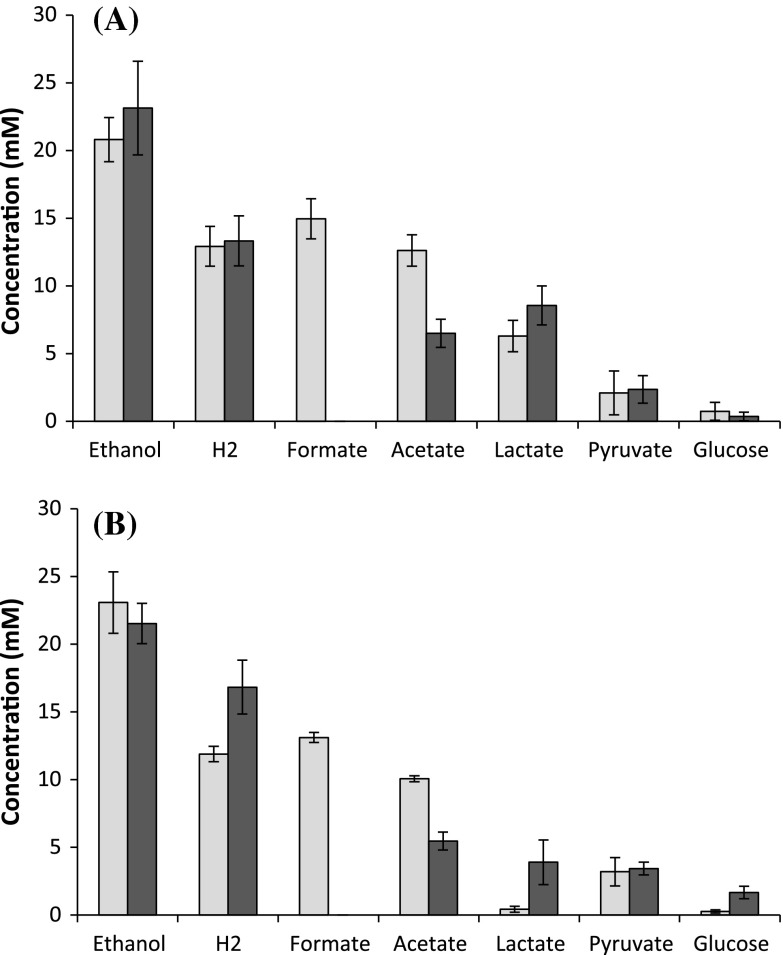
Final fermentation products of *C. thermocellum* strains on **a** complex medium (CTFUD) and **b** defined medium (MTC_5_). All fermentation products were measured upon completion of cellobiose utilization. *Light grey bars*, Δ*hpt*; *Dark grey bars*, Δ*pfl*

*C. thermocellum* Δ*hpt* and Δ*pfl* were grown on 4.5 g/l (13.1 mM) cellobiose in complex (CTFUD) medium or defined medium (MTC_5_). Final fermentation products were measured following nearly complete fermentation of cellobiose (<0.25 mM remaining). Deletion of *pfl* eliminated formate production and decreased acetate production by ~50 %, regardless of medium used (Fig. 2). However, there were significant medium-dependent differences in other final fermentation products. On CTFUD, no differences were observed in final H_2_, ethanol, or lactate concentrations in Δ*pfl* cultures when compared to Δ*hpt*. However, when *C. thermocellum* was grown on MTC_5_, Δ*pfl* cultures produced 1.4-fold more H_2_ and 9.3-fold more lactate compared to the wild type along with comparable concentrations of ethanol. Notably, Δ*hpt* cultures grown on CTFUD produced 15-fold more lactate than on MTC_5_. Residual glucose was detected at the end of growth in all cultures and ranged from 0.3 mM in Δ*hpt* cultures grown on MTC_5_ to 1.7 mM in Δ*pfl* cultures grown on MTC_5_ (Fig. 2, Online Resource 3). Secreted pyruvate was also detected on CTFUD (2.1 and 2.4 mM) and MTC_5_ (3.2 and 3.4 mM) in Δ*hpt* and Δ*pfl* cultures, respectively (Fig. 2, Online Resource 3).

Given that the addition of sodium bicarbonate to MTC_5_ rendered accurate measurements of CO_2_ infeasible, CO_2_ was calculated based on detected fermentation products (Table 1). As expected, deletion of *pfl* resulted in an increase in calculated CO_2_ production of 1.6- and 1.3-fold on CTFUD and MTC_5_, respectively. Oxidation/reduction balances (O/R) based on formate, ethanol, H_2_, and calculated CO_2_ were ~ 1, in support of the validity of our carbon accounting assumptions.

**Table 1:** Final product yields and fermentation balances of Δ*hpt* and Δ*pfl* on CTFUD and MTC

	Product yields (mol mol-hexose consumed^−1^)						O/R balance^b^	C recovery (%)^c^			e^−^ recovery (%)^c^		
	Ethanol	H_2_	Formate	Acetate	Lactate	CO_2_^a^		FPs	AAs	Total	FPs	AAs	Total
CTFUD													
Δ*hpt*	0.77	0.48	0.56	0.47	0.23	0.74	1.05	84.5	1.6	86.2	83.7	1.1	85.9
Δ*pfl*	0.87	0.50	0.00	0.25	0.32	1.19	1.09	79.3	−0.7	78.5	77.5	−0.1	77.5
MTC													
Δ*hpt*	0.87	0.45	0.50	0.38	0.02	0.83	1.04	72.5	7.7	80.2	71.8	9.0	80.8
Δ*pfl*	0.81	0.63	0.00	0.21	0.15	1.07	1.01	73.4	3.4	76.8	73.3	4.2	77.5

Carbon recoveries of fermentation products (FPs), amino acids (AAs), and total secreted products are provided

^a^CO_2_ was calculated using C_1_:C_2_ ratio using ethanol, acetate and formate concentrations

^b^O/R balance was determined using formate, calculated CO_2_ (from C1:C2 ratio), H_2_, and ethanol

^c^Carbon and electron recoveries were calculated based on all substrates (cellobiose and glucose) and end-products (ethanol, H_2_, formate, acetate, lactate, pyruvate) detected, calculated CO_2_ (from C_1_:C_2_ ratio), and secreted amino acids, but exclude biomass measurements

### Select amino acids are utilized by *C. thermocellum*

**Fig. 3:**
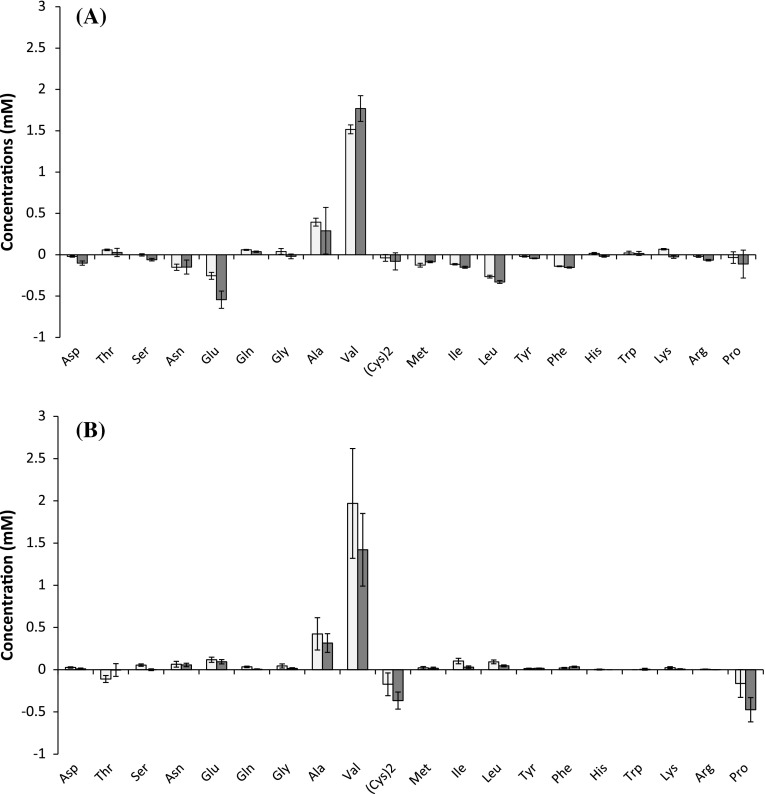
Final secreted amino acid concentrations of *C. thermocellum* strains on **a** complex medium (CTFUD) and **b** defined medium (MTC_5_). All fermentation products were measured upon completion of cellobiose utilization. *Light grey bars* Δ*hpt*; *Dark grey bars* Δ*pfl*; (Cys)2, cystine. *Negative values* indicate net consumption of the given amino acid. *Error bars* represent one standard deviation from the mean

Changes in medium amino acid concentrations at the end of growth are depicted in Fig. 3. Valine and alanine were secreted at the highest levels, regardless of medium or strain used. For CTFUD, which contains yeast extract (Online Resource 3), glutamate and leucine were consumed by both Δ*hpt* (0.4 and 0.3 mM, respectively) and Δ*pfl* (0.7 and 0.3 mM, respectively). All other amino acids were consumed or produced at concentrations less than 0.15 mM (Fig. 3a) on CTFUD. Secreted valine concentrations were 1.5 and 1.8 mM, while secreted alanine concentrations were 0.4 and 0.3 mM, respectively, for Δ*hpt* and Δ*pfl* on CTFUD. While MTC_5_ was not supplemented with amino acids other than cysteine (used as a reducing agent), 1.4 mM proline and 0.3 mM threonine were detected in uninoculated MTC_5_. Consumption of both cysteine and proline was observed by both strains in MTC, albeit consumption of each was greater by Δ*pfl* (Fig. 3b). While final concentrations of valine were 2.0 and 1.4 mM for Δ*hpt* and Δ*pfl*, respectively, and those for alanine were 0.4 and 0.3 mM for Δ*hpt* and Δ*pfl*, respectively, concentrations of all other secreted amino acids were less than 0.15 mM.

Total carbon and electron recoveries in fermentation products were similar between strains under each growth condition and ranged from 77 to 86 % (Table 1). Net production of amino acids accounted for ca. 2 % of final carbon and electron balances in CTFUD-grown Δ*hpt* supernatants, whereas no net difference in amino acids was observed in CTFUD-grown Δ*pfl* supernatants. On MTC_5_, however, total secreted amino acids accounted for ca. 8 % of carbon and electron recoveries in Δ*hpt* supernatants, and ca. 4 % in Δ*pfl* supernatants (Table 1, Online Resource 3).

### Growth rate is altered in *C. thermocellum* Δ*pfl*

**Fig. 4:**
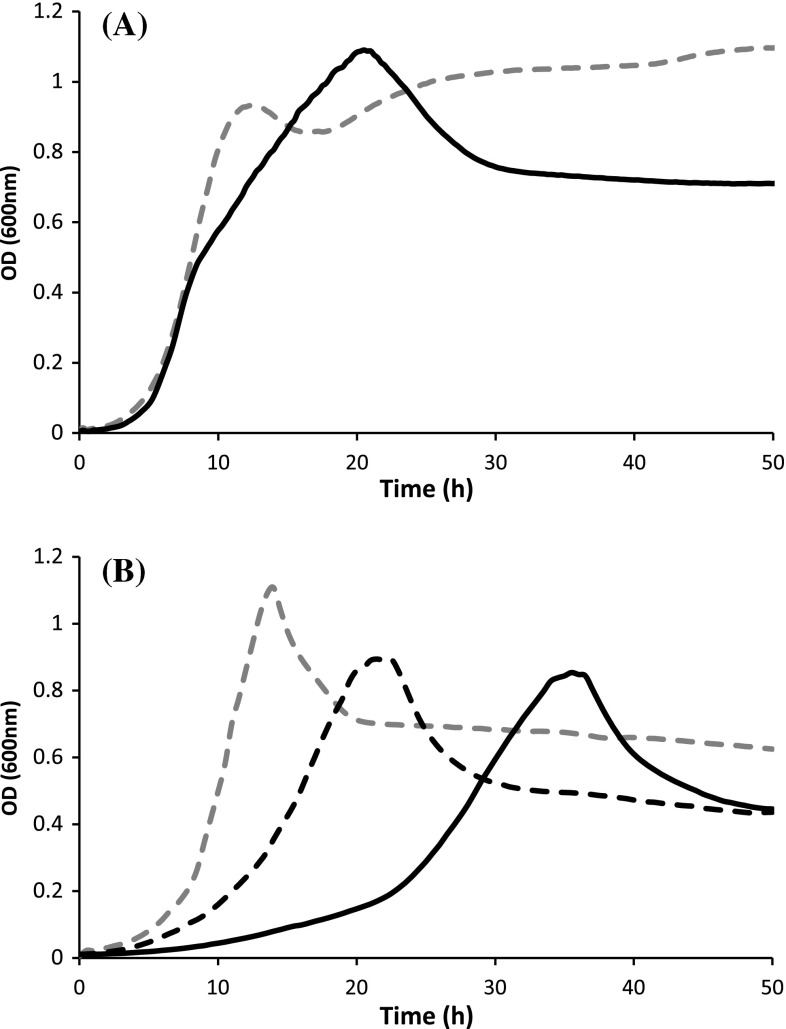
Growth profiles of parent and mutant strains on **a** complex medium (CTFUD) and **b** defined medium (MTC_5_). *Dashed grey line* Δ*hpt*; *solid black line* Δ*pfl*; *dashed black line* Δ*pfl* supplemented with 2 mM formate

To further examine the effect of *pfl* deletion in *C. thermocellum*, we examined growth of the parent strain and the Δ*pfl* mutant on cellobiose in CTFUD and MTC_5_. Growth of the parent strain and Δ*pfl* were similar on complex CTFUD for the first 7 h, with an exponential-phase generation time of 1.3 h (±0.1) and 1.2 h (±0.2), respectively (Fig. 4a). At an optical density of ca. 0.5, Δ*pfl* began to grow slower [9.3 h (±2.7) doubling time] than the parent strain for an additional 10 h until growth peaked at an OD_600_ of 1.1. The final pH of Δ*hpt* and Δ*pfl* cultures was 5.9 and 6.3, respectively.

On defined medium MTC_5_, both Δ*hpt* and Δ*pfl* exhibited a typical-shaped growth curve (Fig. 4b). While Δ*hpt* grew to the same maximum OD_600_ (1.1) on defined medium when compared to CTFUD, its exponential-phase generation time increased to 2.0 h (±0.4). Deletion of *pfl* almost tripled the generation time to 6.0 h (±1.6) and marginally decreased maximum OD_600_ to 0.9 when compared to the parent strain. The final pH of Δ*hpt* and Δ*pfl* cultures in MTC_5_ medium was 7.0 and 6.7, respectively. In *Staphylococcus aureus*, growth of a *pfl* mutant was improved by addition of 2 mM formate [17]. Therefore, we supplemented MTC_5_ with 2 mM formate, which decreased the generation time of Δ*pfl* to 3.4 (±0.5) but had negligible impact on maximum OD_600_.

## Discussion

Improvement of *C. thermocellum* ethanol yields may be achieved through elimination of branched fermentation pathways that divert carbon and electron flux away from ethanol. Previous reports have demonstrated that deletion of genes responsible for acetate [1, 39], lactate [1, 2], and H_2_ synthesis [3] can increase ethanol yields. Production of formate, which has also been shown to be a major fermentation product of *C. thermocellum* [37], can also reduce the amount of electrons that are available for ethanol production [31]. Deletion of *pfl* completely eliminated formate production, demonstrating that it encodes the only functional pathway to formate synthesis in *C. thermocellum* DSM 1313.

The amount of formate produced by *C. thermocellum* varied considerably in previous studies and comparisons are complicated by differences in medium composition, pH control, headspace gas accumulation, carbon loading, and batch vs. continuous fermentation. Typical formate yields in *C. thermocellum* DSM 1313 wild type ranges from 0.05 to 0.15 mol mol-hexose^−1^ [11, 41, 44] but can be as high as 0.27 mol mol-hexose^−1^ in Δ*hpt* cultures [2]. In wild type *C. thermocellum* strain ATCC 27405, reported formate yields range from 0.07 to 0.48 [5, 8, 31, 32]. Here we observed formate yields of 0.57 and 0.50 mol mol-glc^−1^ for wild-type strains on CTFUD and MTC_5_, respectively, both of which were on the higher end of the spectrum when compared to previous results. This variability in medium and/or strain-dependent yields could prove problematic for applied processes; however, by deleting *pfl*, this variable flux to formate is eliminated during *C. thermocellum* cellulose fermentations.

In the absence of *pfl*, conversion of pyruvate to acetyl-CoA is only catalyzed by pyruvate:ferredoxin (Fd) oxidoreductase (PFOR), which generates CO_2_ and reduced Fd (Fig. 1). Calculated final CO_2_ concentrations were higher in Δ*pfl* when compared to Δ*hpt* by 1.6 and 1.4-fold on CTFUD and MTC_5_, respectively, suggesting that carbon and electron flux through PFOR is increased. This is in agreement with previous studies in which PFL activity in *C. thermocellum* ATCC 27405 was inhibited using hypophosphite [31]. Despite the increase of additional reducing equivalents produced in the form of reduced Fd, changes in ethanol production were minimal, whereas acetate production decreased by ca. 50 %, regardless of medium used. Consequently, the increase in calculated CO_2_ production was only 76 and 49 % of the decrease in formate production on CTFUD and MTC_5_, respectively, demonstrating that increased flux through PFOR does not fully compensate for loss of *pfl*, and reduced overall flux from pyruvate to acetyl-CoA may lead to a build-up of intermediates upstream of acetyl-CoA that are diverted away from acetate and ethanol.

On MTC_5_, deletion of *pfl* increased carbon and electron flux towards lactate and H_2_ and decreased carbon flux towards acetate. Given that lactate dehydrogenase requires fructose-1,6-bisphosphate (FbP) as an allosteric activator [28, 32], it may be that higher lactate production in the Δ*pfl* strain is a result of FbP accumulation due to restrictions on the rate of glycolytic flux when conversion of pyruvate to acetyl-CoA is catalyzed by PFOR without PFL. Surprisingly, ethanol production did not increase in the Δ*pfl* strain, consistent with potential limitation by acetyl-CoA or NADH availability. On CTFUD, only a marginal increase in lactate and ethanol was observed, while H_2_ production did not change, suggesting that carbon and electron flux is diverted elsewhere. The two most abundant secreted amino acids, valine and alanine, are derived from pyruvate; therefore, one might hypothesize that the reduced flux from pyruvate to acetyl-CoA might increase flux to secreted amino acids. However, amino acid secretion in fact decreased in Δ*pfl* cultures when compared to Δ*hpt*, regardless of medium used, suggesting that carbon and electron flux is diverted elsewhere.

**Fig. 5:**
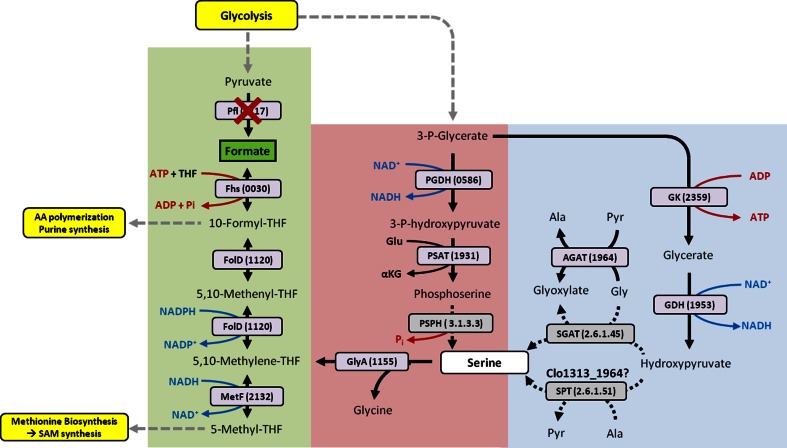
Proposed formyl-THF synthesis pathway in the presence and absence of formate. Formyl-THF may be synthesized directly from formate via formate-THF ligase (Fhs). In the absence of formate, we propose that serine is a precursor for formyl-THF synthesis. While no annotated phosphoserine phosphatase (PSPH) or serine-pyruvate aminotransferase (SPT) is annotated in *C.thermocellum*, alanine-glyoxylate transaminase (AGAT) may function as a SPT. Annotated gene numbers (Clo1313) are indicated in *brackets*. Enzyme E.C. numbers are provided for enzymes which are not annotated in *C. thermocellum*. *THF* tetrahydrofolate; *GK* glycerate kinase; *GDH* glycerate dehydrogenase; *PGDH* 3-phosphoglycerate dehydrogenase; *PSAT* phosphoserine aminotransferase); *SGAT* serine-glyoxylate aminotransferase; *GlyA* serine hydroxymethyltransferase; *MetF* methylene-THF reductase; *FolD* bifunctional methylene-THF dehydrogenase/methylene-THF cyclohydrolase; *Pfl* pyruvate:formate lyase

While deletion of *pfl* eliminated formate production, growth of the Δ*pfl* strain was hindered, especially in minimal medium. On MTC_5_ the exponential phase growth rate of the Δ*pfl* strain was reduced to 40 % of that of the parent strain, and final OD_600_ decreased by 21 %. Given that the Δ*pfl* strain produced ~50 % less acetate, a decrease in ATP availability could partially explain lower final growth yields. Interestingly, while deletion of *pfl* could potentially decrease metabolic flux upstream of acetyl-CoA and, in turn, decrease growth rate, exponential phase growth rate was restored to 80 % of that of the parent strain when MTC_5_ was supplemented with 2 mM formate. Similar restoration of growth rate was observed with formate supplementation for a *Staphylococcus aureus* Δ*pfl* mutant [17]. Formate is commonly used for formyl-tetrahydrofolate (THF) synthesis via formate-THF ligase (Clo1313_0030). Formyl-THF in turn acts as a key donor of formyl groups required for purine and formylmethionine synthesis, as well as methyl group donor for synthesis of methionine and S-adenosyl-methionine (Fig. 5). Thus, we hypothesize that elimination of formate production in *C. thermocellum* has an adverse impact on C_1_ metabolism, causing a growth defect. Despite this, the Δ*pfl* strain is able to grow in minimal medium without formate supplementation, albeit poorly, indicating that *C. thermocellum* has an alternate route for synthesizing formyl- and methyl-THF. In the absence of formate supplementation or synthesis via PFL, we postulate that serine may be a precursor for formyl-THF synthesis (Fig. 5). While genomic analysis reveals the presence of all enzymes required for formyl-THF synthesis from serine in *C. thermocellum*, the pathway for serine biosynthesis is unclear. Possible routes could include phosphoserine phosphatases (PSPH) or serine-pyruvate transaminases (SPT), but none are annotated in *C. thermocellum*. One possibility is that PSPH activity is present in *C. thermocellum* but is encoded by an uncharacterized phosphatase. Alternatively, the annotated alanine-glyoxylate transaminase (AGAT) may have SPT activity, synthesizing serine from hydroxypyruvate. Indeed, other studies have demonstrated that the *Arabidopsis* AGAT can have both AGAT and SPT activities [18, 19]. Regardless of the pathway used to make formyl- and methyl-THF in Δ*pfl*, this pathway is clearly less efficient than the native pathway utilizing formate. Future improvement in formyl- and methyl-THF synthesis in the absence of *pfl* will likely improve the growth rate of these strains.

## Conclusion

In this study, we have eliminated formate production in *C. thermocellum* by deleting genes encoding PFL and PFL-activating enzyme. Redirecting metabolic flux away from PFL towards PFOR could have generated additional electrons available for ethanol production. Although ethanol yields did not increase, other fermentation products that act as electron sinks (i.e., H_2_ and lactate) did increase. Thus, future strategies to improve ethanol yields may involve deletion of lactate and H_2_-formation pathways in conjunction with deletion of PFL to further limit electron flux towards ethanol. Here we demonstrate that formate availability is important in maintaining growth rate in *C. thermocellum* and propose that it acts as a precursor for methyl- and formyl-THF biosynthesis, and ultimately methionine, purine and formylmethionine synthesis. Thus, future improvement in formyl-THF synthesis in the absence of *pfl* will likely improve the growth rate of these strains, which will be required to reduce energy inputs and costs of large-scale fermentations.

## Electronic supplementary material

Supplementary material 1 (DOCX 44 kb) Online Resource 1 Key elements of pAMG281 used for deletion of PFL. CEN6/ARSH4 origin, yeast origin of replication; URA3, orotidine 5′-phosphate decarboxylase; bla, beta-lactamase; pNW33 N origin, *C. thermocellum* origin of replication; P-cbp, *C. thermocellum* cellobiose phosphorylase promoter; tdk, thymidine kinase; pUC origin, *E. coli* origin of replication; CYC1 term, *Saccharomyces cerevisiae* cytochrome C1 transcriptional terminator; pfl up, upstream sequence of homology to *pflA*; pfl down, downstream sequence of homology to *pflB*; P-gapD, *C. thermocellum* glyceraldehyde-3-phosphate dehydrogenase promoter; cat, chloramphenicol acetyltransferase; hpt, hypoxanthine phosphoribosyltransferase; t1t2, T1-T2 terminator; pfl internal, internal sequence of homology to *pflB*. Full plasmid sequence can be obtained by accessing GenBank accession number KP864661

Supplementary material 2 (DOCX 385 kb) Online Resource 2 Deletion of *pfl* and *pfl*-*AE*; overview and confirmation. A) The *pfl* and *pfl*-*AE* locus was deleted according to the protocol outlined by Argyrose et al. (2011). Primer binding sites used to amplify *pfl* fragments (P1 and P2) and the locus encompassing *pfl* and *pfl*-*AE* (P3), and corresponding expected product sizes in the parent and mutant strain are indicated. B) PCR confirmation of *pfl* and *pfl*-*AE* deletion. Primer sets P1 and P2 amplified 705 bp and 707 bp fragments of the *pfl* gene, respectively, in the Δ*hpt* strain, but not in Δ*pfl*. Primer set P3 amplified the chromosomal region that includes *pfl*, and results in a 5116 bp fragment in the parent strain and a 2105 bp fragment in Δ*pfl*, confirming deletion of *pfl*. Absence of P1 and P2 amplicons and a reduction in size of the P3 amplicon confirm deletion of *pfl* and *pfl*-*AE* in the mutant strain

Supplementary material 3 (XLSX 50 kb) Online Resource 3 Fermentation product, amino acid, and growth data with corresponding product yields and O/R, carbon, and electron balances
