# Supplementary material for: Elimination of formate production in Clostridium thermocellum
Source: J Ind Microbiol Biotechnol. 2015 Jul 11;42(9):1263–72. doi: 10.1007/s10295-015-1644-3 (PMC4536278; doi:10.1007/s10295-015-1644-3)
Supplement: Supplementary file 1 — Supplementary material 1 (DOCX 44 kb) Online Resource 1 Key elements of pAMG281 used for deletion of PFL. CEN6/ARSH4 origin, yeast origin of replication; URA3, orotidine 5′-phosphate decarboxylase; bla, beta-lactamase; pNW33 N origin, C. thermocellum origin of replication; P-cbp, C. thermocellum cellobiose phosphorylase promoter; tdk, thymidine kinase; pUC origin, E. coli origin of replication; CYC1 term, Saccharomyces cerevisiae cytochrome C1 transcriptional terminator; pfl up, upstream sequence of homology to pflA; pfl down, downstream sequence of homology to pflB; P-gapD, C. thermocellum glyceraldehyde-3-phosphate dehydrogenase promoter; cat, chloramphenicol acetyltransferase; hpt, hypoxanthine phosphoribosyltransferase; t1t2, T1-T2 terminator; pfl internal, internal sequence of homology to pflB. Full plasmid sequence can be obtained by accessing GenBank accession number KP864661 [file 10295_2015_1644_MOESM1_ESM.docx]

**Elimination of Formate Production in *Clostridium thermocellum***

*Journal of Industrial Microbiology and Biotechnology*

Thomas Rydzak^1,2^, Lee R. Lynd^2,3^, Adam M. Guss^1,2*^

^1^Biosciences Division, Oak Ridge National Laboratory, Oak Ridge, Tennessee, United States of America

^2^BioEnergy Science Center, Oak Ridge National Laboratory, Oak Ridge, Tennessee, United States of America

^3^Thayer School of Engineering at Dartmouth College, Hanover, New Hampshire, United States of America

*Correspondence should be addressed to A.M.G: One Bethel Valley Road, Oak Ridge, TN 37831-6038 USA. Fax: + 1-865-576-8646. Email: [gussam@ornl.gov](mailto:gussam@ornl.gov)


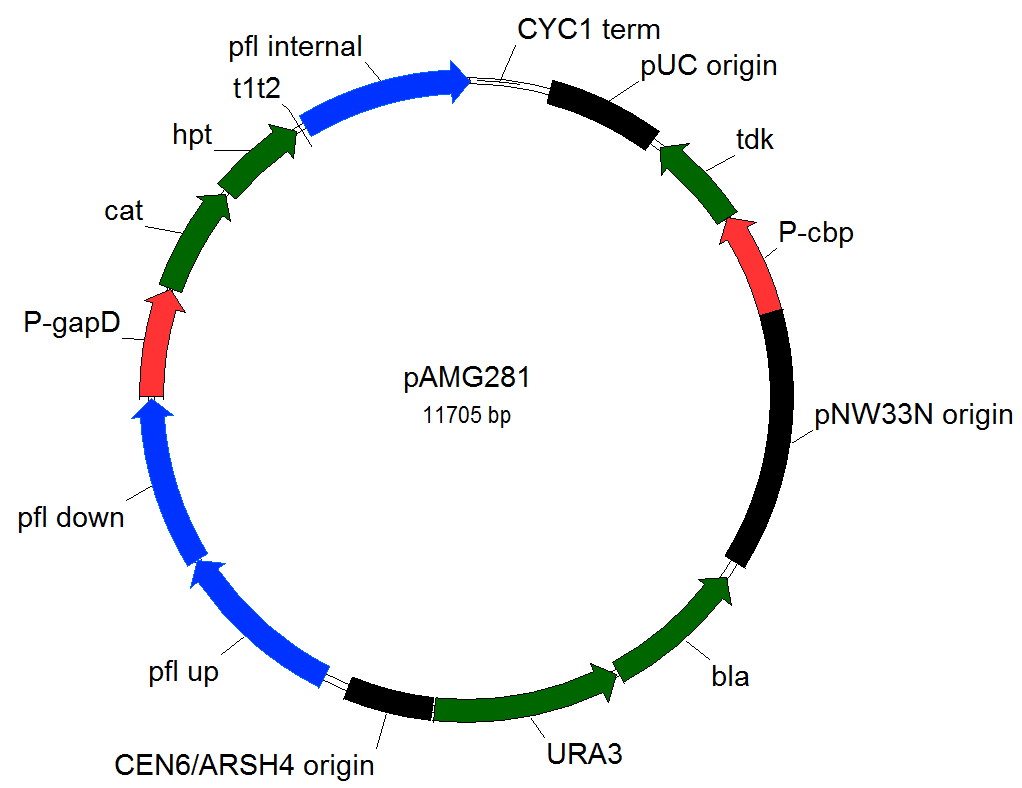


**Online Resource 1:** Key elements of pAMG281 used for deletion of PFL. CEN6/ARSH4 origin, yeast origin of replication; URA3, orotidine 5’-phosphate decarboxylase; bla, beta-lactamase; pNW33N origin, *C. thermocellum* origin of replication; P-cbp, *C. thermocellum* cellobiose phosphorylase promoter; tdk, thymidine kinase; pUC origin, *E. coli* origin of replication; CYC1 term, *Saccharomyces cerevisiae* cytochrome C1 transcriptional terminator; pfl up, upstream sequence of homology to *pflA*; pfl down, downstream sequence of homology to *pflB*; P-gapD, *C. thermocellum* glyceraldehyde-3-phosphate dehydrogenase promoter; cat, chloramphenicol acetyltransferase; hpt, hypoxanthine phosphoribosyltransferase; t1t2, T1-T2 terminator; pfl internal, internal sequence of homology to *pflB*. Full plasmid sequence can be obtained by accessing GenBank accession number KP864661
