# Supplementary material for: Elimination of formate production in Clostridium thermocellum
Source: J Ind Microbiol Biotechnol. 2015 Jul 11;42(9):1263–72. doi: 10.1007/s10295-015-1644-3 (PMC4536278; doi:10.1007/s10295-015-1644-3)
Supplement: Supplementary file 2 — Supplementary material 2 (DOCX 385 kb) Online Resource 2 Deletion of pfl and pfl-AE; overview and confirmation. A) The pfl and pfl-AE locus was deleted according to the protocol outlined by Argyrose et al. (2011). Primer binding sites used to amplify pfl fragments (P1 and P2) and the locus encompassing pfl and pfl-AE (P3), and corresponding expected product sizes in the parent and mutant strain are indicated. B) PCR confirmation of pfl and pfl-AE deletion. Primer sets P1 and P2 amplified 705 bp and 707 bp fragments of the pfl gene, respectively, in the Δhpt strain, but not in Δpfl. Primer set P3 amplified the chromosomal region that includes pfl, and results in a 5116 bp fragment in the parent strain and a 2105 bp fragment in Δpfl, confirming deletion of pfl. Absence of P1 and P2 amplicons and a reduction in size of the P3 amplicon confirm deletion of pfl and pfl-AE in the mutant strain [file 10295_2015_1644_MOESM2_ESM.docx]

**Elimination of Formate Production in *Clostridium thermocellum***

*Journal of Industrial Microbiology and Biotechnology*

Thomas Rydzak^1,2^, Lee R. Lynd^2,3^, Adam M. Guss^1,2*^

^1^Biosciences Division, Oak Ridge National Laboratory, Oak Ridge, Tennessee, United States of America

^2^BioEnergy Science Center, Oak Ridge National Laboratory, Oak Ridge, Tennessee, United States of America

^3^Thayer School of Engineering at Dartmouth College, Hanover, New Hampshire, United States of America

*Correspondence should be addressed to A.M.G: One Bethel Valley Road, Oak Ridge, TN 37831-6038 USA. Fax: + 1-865-576-8646. Email: [gussam@ornl.gov](mailto:gussam@ornl.gov)

**A)**


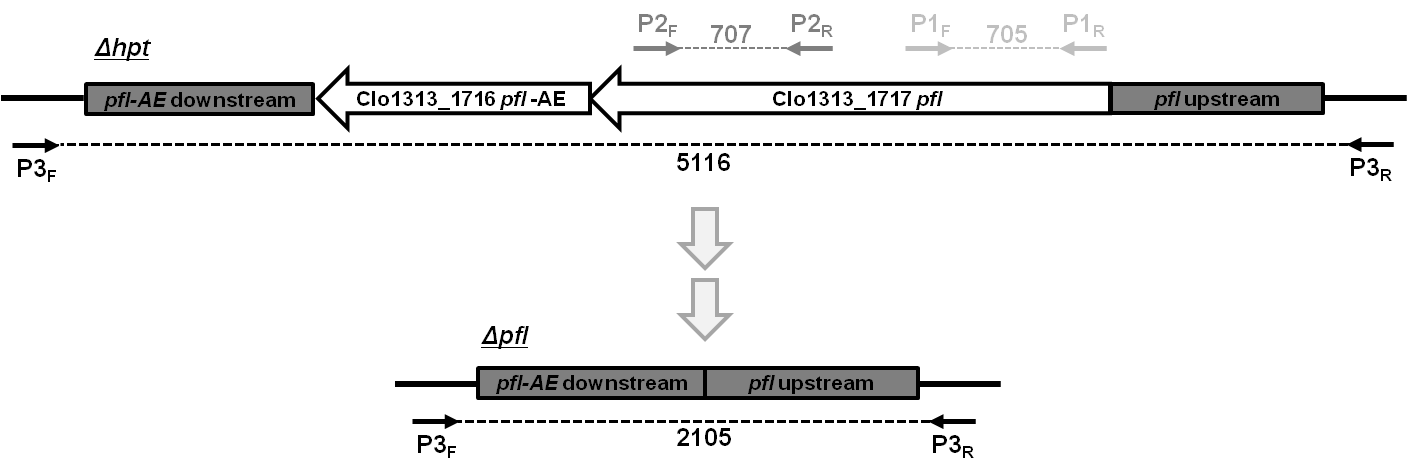


**B)**


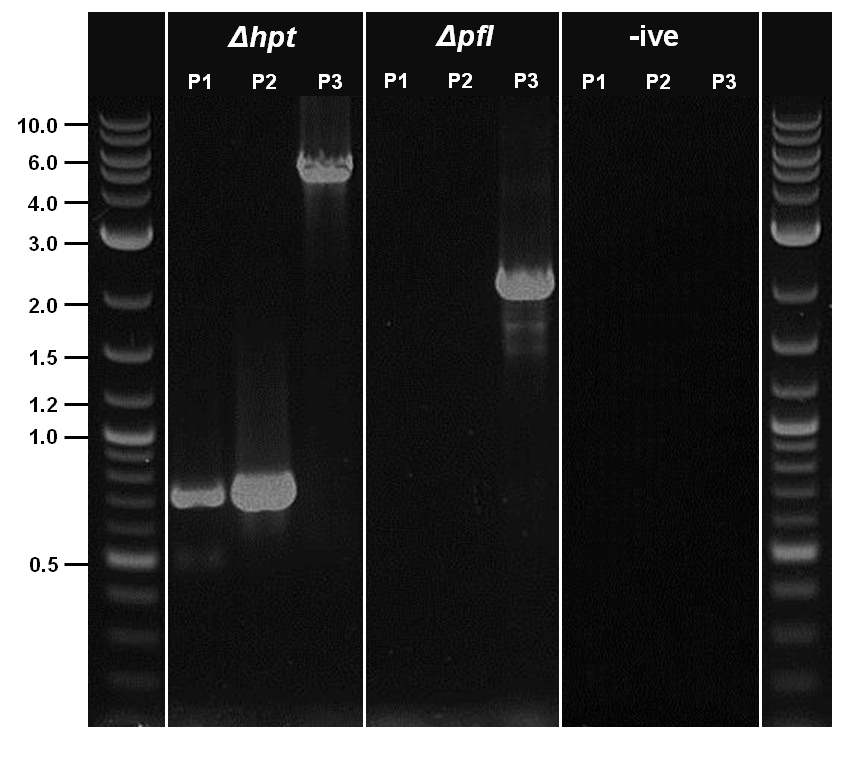


**Online Resource 2:** Deletion of *pfl* and *pfl-AE*; overview and confirmation. A) The *pfl* and *pfl*-*AE* locus was deleted according to the protocol outlined by Argyrose *et al*. (2011). Primer binding sites used to amplify *pfl* fragments (P1 and P2) and the locus encompassing *pfl* and *pfl-AE* (P3), and corresponding expected product sizes in the parent and mutant strain are indicated. B) PCR confirmation of *pfl* and *pfl-AE* deletion. Primer sets P1 and P2 amplified 705 bp and 707 bp fragments of the *pfl* gene, respectively, in the Δ*hpt* strain, but not in *Δpfl*. Primer set P3 amplified the chromosomal region that includes *pfl*, and results in a 5116 bp fragment in the parent strain and a 2105 bp fragment in Δ*pfl*, confirming deletion of *pfl*. Absence of P1 and P2 amplicons and a reduction in size of the P3 amplicon confirm deletion of *pfl* and *pfl-AE* in the mutant strain
